# Supplementary material for: Developing a stakeholder-informed social responsibility model for translational science
Source: PLoS One. 2025 Jun 9;20(6):e0320956. doi: 10.1371/journal.pone.0320956 (PMC12148177; doi:10.1371/journal.pone.0320956)
Supplement: S2 File — (PDF) [file pone.0320956.s002.pdf]

Thanks for Joining Us Today!

Before we start:

Please click the link in the chat or use the QR Code below to complete a brief demographic survey.

1

1

Developing Best Practices related to Social Responsibility of Translational Science

Dr. Grace A. Loudd
Associate Professor, Department of Social Work
Texas Southern University

Dr. Elise Smith
Assistant Professor, Department of Bioethics and Health Humanities
School of Public and Population Health, University of Texas Medical Branch

utmb Health

2

The Problem with Translation

- Historically, scientists often aimed to discover knowledge that may serve to benefit human health.
- However, this knowledge often did not "translate" into improved human health outcomes.

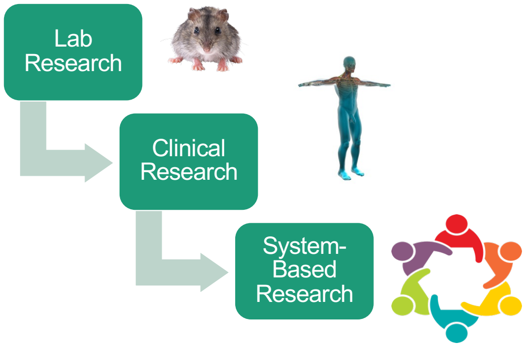

3

3

Perceptions

- What do you know about the concept of social responsibility?
- What **<u>ideals</u>** regarding social responsibility do you believe to be most important and why?**
- What, if anything, limits your perceptions of engaging research that maximizes your social responsibility?

4

4

Translational Science

- Translation is the process of turning observations in the laboratory, clinic, and community into interventions that improve the health of individuals and the public.
- Translational research requires scientists to make earnest efforts to *translate their findings into clinical or population-level applications*.

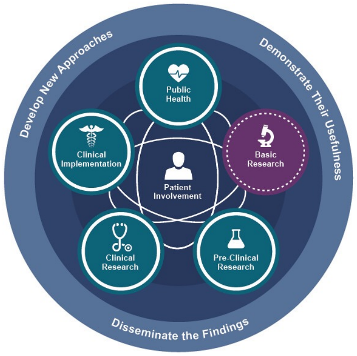

Figure Credit: National Center for Advancing Translational Sciences : https://ncats.nih.gov/translation/spectrum

5

5

Social Responsibility of Translational Science

- There is a ***social responsibility*** within the translational framework to contribute to:
  - Improvement of health benefits
  - Reduction of disparities
- Even if you don't see yourself as a translational researcher, your research likely contributes to the translational pathway.

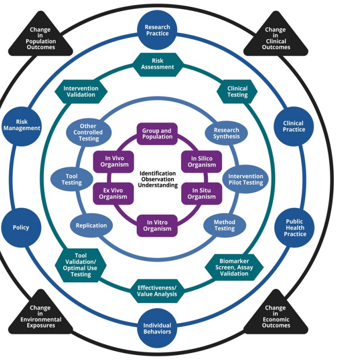

Figure Credit: National Institute for Environmental Health Sciences, https://www.niehs.nih.gov/research/programs/translational/framework-details/index.cfm

6

6

Expectations

- How might you describe existing expectations between scientific research as a whole and its larger social benefit?
- What do you expect the public's role(s) to be within the design, trajectory, and implementation of scientific research?

Expectations**

- Existing literature about the interpretation and definitions of social responsibility in research include six key themes. Based on your personal expectations, experiences, and preferences, select your top 3 themes and place those in the chat.
  1. Consideration of societal consequences;
  2. Protection of human welfare and safety;
  3. Promotion of environmental sustainability;
  4. Efforts to minimize risk;
  5. Communication with the public; and
  6. Services and community engagement.
  7. Other

Expectations

- What, if anything, places limits around your own expectations when it comes to maximizing aspects of social responsibility **within your own research**?

9

9

Behaviors

- What, if any, research-related activities, methods, approaches do you engage in that align with your concept of social responsibility?
- At an institutional level, what research-related activities should be developed and implemented to promote social benefit.**
- What, if anything, limits the range of research activities/behaviors engaged that may align with maximizing social responsibility?

10

10

Commitments

- What are some activities/behaviors/processes you are willing to incorporate and/or implement in your research, if any, that enhances aspects of social responsibility?**

- What do you foresee as **primary barriers** to executing or maintaining any of the commitments identified?

11

11

Close-out

- As we conclude, we'd like to ask you to individually share any thoughts or reflections about any aspect of today's conversation.

  - For example, compared to the beginning of our discussion, how might your **perception** of or **expectations** around social responsibility changed, if at all?

12

12
