# Supplementary material for: Developing a stakeholder-informed social responsibility model for translational science
Source: PLoS One. 2025 Jun 9;20(6):e0320956. doi: 10.1371/journal.pone.0320956 (PMC12148177; doi:10.1371/journal.pone.0320956)
Supplement: S3 File — (PDF) [file pone.0320956.s003.pdf]

Thanks for Joining Us Today!

Before we start:

Please click the link in the chat or use the QR Code below to complete a brief demographic survey.

1

1

Developing Best Practices related to Social Responsibility of Translational Science

Dr. Grace A. Loudd
Associate Professor, Department of Social Work
Texas Southern University

Dr. Elise Smith
Assistant Professor, Department of Bioethics and Health Humanities
School of Public and Population Health, University of Texas Medical Branch

utmb Health

2

Perceptions

- What do think of when you hear the word social responsibility?
- What **<u>aspects of social responsibility</u>** do you find most important and why?**

3

The Problem with Translation

- Historically, scientists often aimed to discover knowledge that may serve to benefit human health.
- However, this knowledge often did not “translate” into improved human health outcomes.

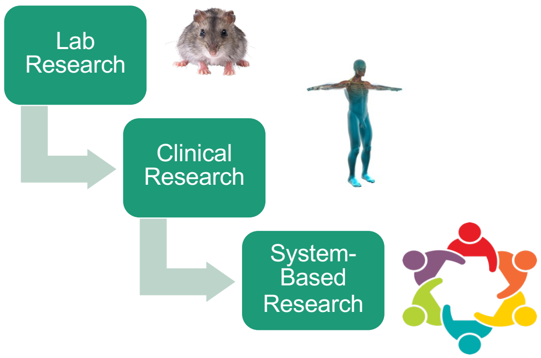

4

Translational Science

- Translation is the process of turning observations in the laboratory, clinic, and community into interventions that improve the health of individuals and the public.
- *The National Center for the Advancement of Translational Science (NCATS) aims to "deliver more treatments to more people more quickly"*

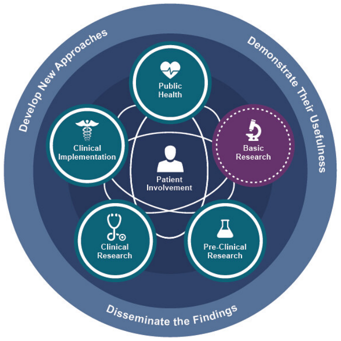

Figure Credit: National Center for Advancing Translational Sciences : https://ncats.nih.gov/translation/spectrum

5

Social Responsibility of Translational Science

- There is a ***social responsibility*** within translational framework to contribute to:
  - Improvement of health benefits
  - Reduction of disparities
- As a community member, your contribution to this framework can take many forms.

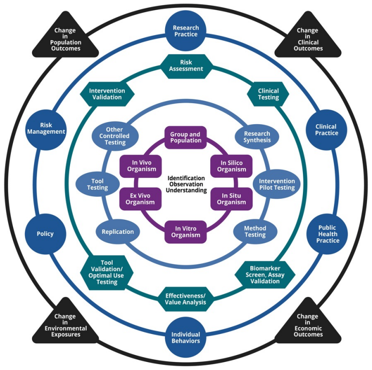

Figure Credit: National Institute for Environmental Health Sciences, https://www.niehs.nih.gov/research/programs/translational/framework-details/index.cfm

6

Expectations

- What are your expectations as to how research benefits communities?
- What do you expect the public's role(s) to be in how research is executed?**

7

7

Expectations

- Existing literature about the interpretation and definitions of social responsibility in research include six key themes. What themes **would you prioritize** based on your personal expectations, experiences, and preferences.

  1. Consideration of societal consequences;
  2. Protection of human welfare and safety;
  3. Promotion of environmental sustainability;
  4. Efforts to minimize risk;
  5. Communication with the public; and
  6. Services and community engagement.
  7. Other

8

8

Behaviors

- What are some examples of **community-based activities or opportunities** that you or someone you know observed/engaged in that align with socially responsible research?

- What are some actions larger organizations, state, and/or federal government could do **to reinforce the benefit of research** on behalf of community?

9

9

Commitments

- Compared to the beginning of our discussion, how might your **perception** of social responsibility changed, if at all?
- Compared to the beginning of our discussion, how might your **expectations** around social responsibility changed, if at all?**
- Do you have any more suggestions or ideas to suggest regarding the social responsibility of science?

10

10
